# Vaccination Coverage and Determinants Among Children Aged 12–35 Months Following Internal Conflict in Yemen: Insights from a Nationwide Population-Based Survey

**DOI:** 10.1007/s10995-025-04156-w

**Published:** 2025-09-01

**Authors:** Omid Dadras, Christina El Saaidi

**Affiliations:** 1 Research Center for Child Psychiatry, University of Turku, Lemmkaisenkatu 3, 20520 Turku, Finland; 2 Metropolitan Health District- The City of San Antonio Texas, San Antonio, USA

**Keywords:** Vaccination coverage, Yemen, Children, Immunization

## Abstract

**Introduction:**

Child vaccination is a crucial public health indicator, especially in conflict-affected regions. Despite the benefits, vaccination rates in Yemen remain suboptimal. This study evaluates vaccination coverage and its correlates among children aged 12–35 months in Yemen.

**Methods:**

Data from the Yemen Multiple Indicator Cluster Survey (MICS) 2022-23 were used. The survey covered 22 governorates, using a two-stage household selection process, and included 7,796 children. Vaccination status was assessed using a binary composite variable for full immunization. Multilevel logistic regression with robust error variance identified predictors of full vaccination.

**Results:**

The overall vaccination coverage was 29%, with urban areas (41%) having higher rates compared to rural areas (25%). Female children had slightly lower odds of being fully vaccinated than male children, though not statistically significant. First-born children had the highest vaccination rates (31%), with odds decreasing with higher birth order. Mothers’ secondary or higher education (AOR: 1.59, 95% CI: 1.19–2.13), receiving prenatal care (AOR: 1.97, 95% CI: 1.26–3.07), and reading newspapers at least once a week (AOR: 1.72, 95% CI: 1.21–2.44) were significant positive predictors. Higher fathers’ education, fewer children under five in the household, higher household wealth, and urban residence were also associated with higher vaccination rates. Other factors such as hospital delivery, TV watching, internet access, and mobile phone ownership were not significantly associated with full vaccination after adjustment.

**Conclusion:**

Significant gaps in immunization coverage among children in Yemen, particularly in rural areas, highlight the need for educational programs for parents, enhanced healthcare infrastructure, and improved health communication strategies.

## Significance

This study sheds light on the critical issue of vaccination disparity in Yemen, underscoring the influence of socioeconomic factors and parental education on immunization rates. It emphasizes the urgency for targeted interventions that address both the systemic barriers and cultural factors hindering vaccination. By highlighting the predictors of full immunization, this research provides a roadmap for policymakers and public health officials to design more effective, localized strategies. Ultimately, it aims to contribute to a broader effort to improve child health outcomes and reduce vaccine-preventable diseases in conflict-affected and resource-limited settings.

## Introduction

Childhood immunization is widely recognized as one of the most effective public health interventions, significantly reducing the incidence of vaccine-preventable diseases and child mortality globally. Vaccination programs have successfully eradicated diseases like smallpox and substantially reduced the prevalence of polio, measles, and diphtheria (UNICEF, 2020; WHO, 2023). Despite these achievements, achieving high immunization coverage remains a significant challenge, particularly in conflict-affected zones (Jelle et al., 2023; Singh et al., 2019). Various factors contribute to lower vaccination rates in these regions, including logistical difficulties, political instability, and socio-economic barriers. Conflict-affected zones often face severe interruptions, such as closures of clinics, shortages of trained staff, and disrupted vaccine supply chains in healthcare services, which leads to increased disease outbreaks due to lower vaccination coverage (de Lima Pereira et al., 2018; Mbaeyi, 2023; Ngo et al., 2020). In addition, damaged infrastructure, poverty-driven inability to afford care, lack of transportation, and overall disruption of health insurance or public funding further limit access to routine immunization services. Population displacement and insecurity compound these challenges, hindering families’ ability to reach even functioning health facilities (Marou et al., 2024).

Since 2015, Yemen has been going through a major war with a devastating influence on healthcare infrastructure with only 45% of health facilities functioning (Torbosh et al., 2019). The ongoing conflict has damaged clinics and hospitals, destroyed roads and bridges, triggered fuel shortages, and created pervasive insecurity along travel routes, making it difficult for families to reach even operational facilities; out-of-pocket costs and breakdowns in public funding have further compounded these barriers (El Bcheraoui et al., 2018; Torbosh et al., 2019). This resulted in a sharp fall in vaccination coverage below herd immunity level. For example, measles vaccine coverage among children 12–23 months decreased from 75% in 2014 to 66% in 2015 at the national level (Torbosh et al., 2019). The decline in vaccination rates led to a surge in vaccine-preventable disease outbreaks such as measles, diphtheria, and cholera among Yemeni children after the start of the war (Qirbi & Ismail, 2016; Raslan et al., 2017). While the conflict has undoubtedly been the primary driver of Yemen’s declining vaccination rates, a more granular understanding of other potential factors could inform targeted interventions and strategies to improve coverage in specific communities or regions, even amidst the ongoing crisis. Although a recent UNICEF report (UNICEF, 2024) described the vaccination coverage among children aged 1–2 years in Yemen, our study extends this work by examining immunization gaps in children up to 35 months of age, providing novel insights into delayed or missed booster doses in older toddlers and their correlates (UNICEF, 2024).

Against this background, this study aims to evaluate vaccination coverage among children aged 12–35 months in Yemen and identify the sociodemographic factors influencing their immunization status using the data from the Yemen Multiple Indicator Cluster Survey (MICS), conducted between 2022 and 2023. By identifying gaps in immunization coverage and understanding the socio-demographic factors that influence vaccination uptake, this study seeks to inform policy and programmatic efforts to improve vaccine delivery in conflict-affected settings. Furthermore, this research contributes to the broader understanding of how sociodemographic factors impact public health interventions, ultimately guiding efforts to enhance child health outcomes in Yemen and achieve global immunization goals.

## Methods

### Study Setting

This study is a secondary analysis of the Yemen MICS, conducted between 2022 and 2023 by the Central Statistical Organization (CSO), with support from the United Nations Children’s Fund (UNICEF). The MICS is a nationally representative, household-based survey that uses a standardized methodology developed by UNICEF to monitor key indicators related to the health, education, and wellbeing of women and children. The publicly available anonymized dataset was accessed through the UNICEF MICS website. The Yemen MICS 2022–2023 survey aimed to gather comprehensive data on the well-being of children and women across the country’s 22 governorates, both in urban and rural settings (Yemen, 2022−23).

## Sampling

**Fig. 1:**
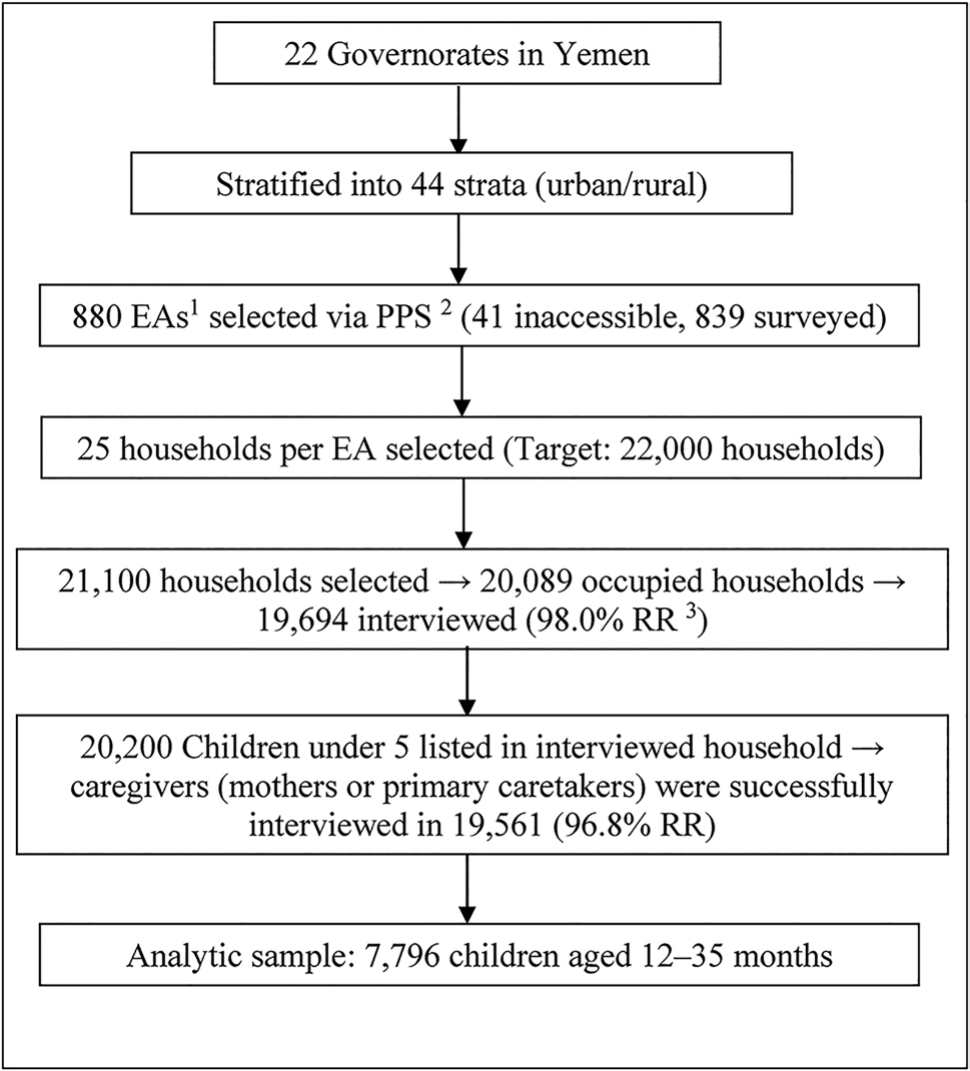
Sampling design (Yemen MICS 2022-23). ^1^EA: Enumeration Area, ^2^PPS: Probability proportional to size, ^3^RR: Repsonse rate

The sample was carefully designed to represent a diverse range of indicators, utilizing a two-stage household selection process. Initially, urban and rural areas within each governorate were identified as primary sampling strata, with census enumeration areas chosen systematically based on their size. Subsequently, 25 households were systematically selected from each sampled enumeration area. Although the target sample size was 880 clusters and 22,000 households, logistical challenges resulted in 41 enumeration areas being inaccessible due to security concerns during fieldwork. Eventually, 21,100 households were selected, of which 20,089 were occupied, and 19,694 were successfully interviewed. For 19,561 out of 20,200 children under 5 listed in included households, the questionnaires were completed by their caregivers (mothers or primary caretakers), reflecting a response rate of 96.8%. In this study, however, we only included children aged 12–35 months, with a total number of 7796. Figure 1 illustrates the sampling design in Yemen MICS 2022-23.

## Study Variables

### Outcome Variable

Fully vaccinated (composite = 1) was coded for children who had received all the following antigens by the time of interview:

- BCG (at birth).
- Polio (three doses: OPV1, OPV2, OPV3 or IPV – counted as “Polio3/IPV”).
- Diphtheria-Tetanus-Pertussis (three doses of Pentavalent vaccine).
- Hepatitis B (three doses, as part of Pentavalent).
- Haemophilus influenzae type b (three doses, as part of Pentavalent).
- Measles–Rubella (two doses: MR1 at 9 months and MR2 at 18 months).

Any child missing one or more of these required antigens/doses was coded as not fully vaccinated (composite = 0). Vaccination status combined data from vaccination cards and caretaker recall: a child was considered to have received a dose if either the card recorded it or the mother/caretaker reported it.

Although the Yemen EPI schedule includes three doses of pneumococcal conjugate vaccine (PCV1–3) and two doses of rotavirus vaccine (RV1–2), the standard WHO “full immunization” indicator (as implemented in MICS) focuses on the core antigens listed above (Yemen, 2022−23). We have therefore excluded PCV and rotavirus from our primary composite to maintain comparability with global and regional benchmarks for full immunization (i.e., BCG, Polio3/IPV, DTP3, HepB3, Hib3, Measles2).

## Independent/Exposure Variables

Based on a comprehensive literature review and leveraging the WHO framework on the factors that might influence the receipt of vaccines among unimmunized children (Sawhney & Favin, 2009), we have included 15 potential variables. These comprise variables related to the child and family including the child’s sex (male, female), birth order (1st,2nd, 3rd, 4th or higher), mother’s age (15–20, 21–34, 35–49 years), mother’s and father’s education (no education, primary, secondary or higher), whether mother received prenatal care (yes, no), where the child has been delivered (home, hospital), place of living (rural, urban), number of children under 5 in the household (one, two or more), and household wealth index (poor, middle, rich) as defined and calculated in Yemen MICS 2022-23 (Yemen, 2022−23); variables related to communication and information including reading newspapers, watching TV, or listening radio (no, less or at least once a week), access to the internet at home (yes, no), and owning a mobile phone (yes, no).

### Statistical Analysis

Descriptive statistics were used to describe the distribution of participants’ characteristics and the prevalence of being fully vaccinated. Since there were mothers with more than one child aged 12–35, we employed multilevel logistic regression with robust error variance to examine the relationship between full vaccination and independent variables, accounting for the clustering effect at the mother level (Tamene et al., 2023). The intercept-only model produced an intraclass correlation coefficient (ICC) of 0.08, indicating that approximately 8% of the variance in full vaccination status is attributable to between-mother differences. This moderate level of clustering supports the use of a multilevel approach to avoid biased standard errors and ensure accurate inference (Hox et al., 2017). Significant variables from univariate logistic regression models (*p* < 0.20) were entered into the multivariable model, consistent with recommendations to avoid prematurely excluding potentially important covariates (Bursac et al., 2008). Model selection followed a backward stepwise approach: at each step, the likelihood-ratio test compared the nested model against one with the candidate variable reintroduced, retaining only those whose removal significantly worsened model fit (*p* < 0.05). In addition, we retained variables with strong theoretical relevance—such as internet access, mobile phone ownership, and TV viewing—based on prior evidence linking them to vaccination coverage (Geweniger & Abbas, 2020; Williams et al., 2024), regardless of their statistical significance. We also compared Akaike Information Criterion (AIC) and Bayesian Information Criterion (BIC) values between nested models, choosing the model with the lowest AIC/BIC at each step to balance fit and parsimony. (Harrell & Harrell, 2001). Multicollinearity was further evaluated using variance inflation factors (VIFs) via the Stata “collin” command, which ranged from 1.01 to 1.60 (mean = 1.24), with no VIF exceeding 5.0, indicating that collinearity alone did not necessitate exclusion of predictors (O’brien, 2007) in the initial analysis. However, father’s education was excluded later from the final adjusted model due to (1) moderate multicollinearity with maternal education and household wealth and (2) lack of significant improvement in model fit when included. Results were presented as frequencies (%), unadjusted and adjusted odds ratios (OR and AOR, respectively), and corresponding 95% confidence intervals (CI). Stata 18 software was used for data analysis, and statistical significance was set at *p* < 0.05.

## Results

### Sample Characteristics

**Fig. 2:**
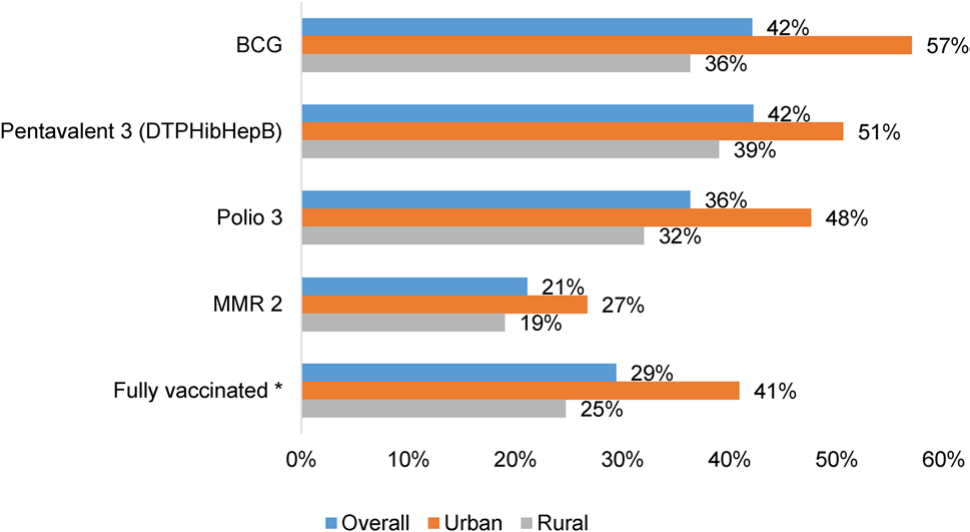
Full vaccination rates among children aged 12–35 months in Yemen; by place of living. *All antigens include: BCG, Polio3/IPV, DTP3, HepB3, Hib3, Rubella and Measles 2 as per the vaccination schedule in Yemen

As Table 1 represents, there were almost equal numbers of boys (50.8%) and girls (49.2%) included in the study. Most children were firstborns (50.9%), followed by secondborns (23.9%), thirdborns (13.4%), and fourth or higher birth order (11.8%). The majority of mothers (66.9%) were between 21 and 34 years old. Nearly 39% of mothers had no education, while 25.9% had primary education, and 35.2% had secondary or higher education. Over two-thirds (67.9%) of mothers received prenatal care. Home delivery (58.0%) was more common than hospital delivery (42.0%). The vast majority (93.1%) of mothers did not report reading newspapers, while a smaller percentage (43.5%) did not watch TV. Over half (54.5%) of households lack internet access, and nearly half (47.4%) do not own a mobile phone. A portion (43.3%) fell under the poor wealth category. Most households (66.9%) had two or more children under 5 years old. The majority of the population (72.0%) lives in rural areas. As Fig. 2 illustrates, the overall vaccine coverage among children aged 12–35 months in Yemen MICS 2022-23 was 29.4%, with urban areas (41%) having a significantly higher rate of full vaccination as compared to rural areas (25%).

**Table 1:** Factors associated with being fully vaccinated among children aged 12–35 months, Yemen MICS 2023

	Total sample	Fully vaccinated		
	N(weighted %)	%	OR (95%)	AOR (95%) ^2^
Sample 12–35 months	7796 (100)	29.4		
Child’s sex				
Male	3965 (50.8)	30.2	Reference	–
Female	3831 (49.2)	28.6	0.94 (0.87, 1.02)	–
Birth order				
1st	2944 (50.9)	31.0	Reference	–
2nd	1471 (23.9)	28.5	0.90 (0.71, 1.16)	–
3rd	821 (13.4)	24.9	0.84 (0.56, 1.28)	–
4th or higher	809 (11.8)	22.0	0.65 (0.48, 0.87)*	–
Mother’s age (year)				
15–20	674 (8.9)	28.9	Reference	–
21–34	5124 (66.9)	30.8	1.00 (0.67, 1.50)	–
35–49	1888 (24.2)	26.8	0.87 (0.52, 1.47)	–
Mother’s education				
No education	3227 (38.8)	20.3	Reference	Reference
Primary	1933 (25.9)	28.9	1.46 (1.32, 1.62)*	1.37 (0.71, 2.62)
Secondary or higher	2632 (35.2)	38.8	2.43 (2.18, 2.72)*	1.59 (1.19, 2.13)*
Received prenatal care				
No	2016 (32.1)	22.8	Reference	Reference
Yes	3245 (67.9)	43.9	2.81 (2.44, 3.23)*	1.97 (1.26, 3.07)*
Place of delivery				
Home	2983 (58.0)	31.7	Reference	Reference
Hospital	2250 (42.0)	44.6	1.73 (1.57, 1.91)*	0.96 (0.70, 1.34)
Read newspaper				
No	2289 (93.1)	20.9	Reference	Reference
Less/at least once a week	204 (6.9)	35.4	2.18 (1.47, 3.23)*	1.72 (1.21, 2.44)*
Watch TV				
No	1135 (43.5)	14.8	Reference	Reference
Less/at least once a week	1351 (56.5)	27.0	2.43 (1.72, 3.44)*	1.07 (0.59, 1.98)
Listen to the radio				
No	2409 (96.3)	21.5	Reference	–
Less/at least once a week	84 (3.7)	27.5	1.07 (0.69, 1.67)	–
Internet access at home				
No	1349 (54.5)	14.3	Reference	Reference
Yes	1193 (45.5)	30.4	2.25 (1.70, 2.97)*	1.31 (0.69, 2.51)
Own a mobile phone				
No	1211 (47.4)	16.0	Reference	Reference
Yes	1280 (52.6)	26.8	1.68 (1.30, 2.18)*	0.84 (0.52, 1.35)
Father’s education				
No education	1102 (13.2)	16.3	Reference	–
Primary	1537 (23.0)	27.4	1.99 (1.65, 2.39)*	–
Secondary	4670 (63.9)	33.2	2.62 (2.19, 3.13)*	–
Number of children under 5				
One	2554 (33.1)	33.3	Reference	Reference
Two or more	52.4 (66.9)	27.5	0.74 (0.69, 0.80)*	0.20 (0.12, 0.34)*
Household’s wealth index				
Poor	3428 (43.3)	22.1	Reference	Reference
Middle	1532 (19.9)	27.5	1.53 (1.29, 1.80)*	1.09 (0.68, 1.74)
Rich	2836 (36.8)	38.5	2.34 (2.12, 2.58)*	2.09 (1.14, 3.83)*
Place of living				
Urban	1860 (28.0)	40.9	Reference	Reference
Rural	5936 (72.0)	24.7	0.46 (0.42, 0.51)*	0.70 (0.51, 0.94)*

*p-value < 0.05. ^1^Note: Totals may not sum to 7796 due to missing values for some variables. ^2^ Variables with univariate association at *p* < 0.20 were entered into a backward stepwise multivariable model; predictors were retained only if their removal significantly worsened model fit (likelihood-ratio test *p* < 0.05) and did not exhibit problematic collinearity (VIF < 5.0)

### Prevalence and Correlates of Full Vaccination Among Children Aged 12–35 Months

The proportion of male children fully vaccinated was 30.2%, compared to 28.6% among females. The odds of being fully vaccinated were slightly lower for female children compared to male children, but this difference was not statistically significant (OR: 0.94, 95% CI: 0.87–1.02). First-born children had the highest vaccination rates (31.0%), followed by second-born (28.5%), third-born (24.9%), and fourth or higher birth order (22.0%). The odds of being fully vaccinated decreased with increasing birth order, with children of the fourth or higher birth order having significantly lower odds (OR: 0.65, 95% CI: 0.48–0.87).

Children of mothers aged 21–34 had a slightly higher vaccination rate (30.8%) compared to those of younger mothers (15–20 years) (28.9%). However, the differences were not statistically significant across maternal age groups. Children of mothers with secondary or higher education were more likely to be fully vaccinated (38.8%) compared to those whose mothers had no education (20.3%). This association remained significant after adjustment (AOR: 1.59, 95% CI: 1.19–2.13).

Among children whose mothers received prenatal care, 43.9% were fully vaccinated, versus 22.8% among those whose mothers did not. Receiving prenatal care was significantly associated with higher odds of being fully vaccinated (AOR: 1.97, 95% CI: 1.26–3.07). Children born in hospitals had higher vaccination rates (44.6%) compared to those born at home (31.7%). However, this association was not significant after adjustment (AOR: 0.96, 95% CI: 0.70–1.34).

Children whose mothers read newspapers at least once a week had a vaccination rate of 35.4%, compared to 20.9% among those whose mothers did not. This association remained significant after adjustment (AOR: 1.72, 95% CI: 1.21–2.44). Watching TV at least once a week was associated with a higher vaccination rate (27.0%) versus 14.8% for those who did not, but this association was not significant after adjustment (AOR: 1.07, 95% CI: 0.59–1.98). Listening to the radio showed no significant association with vaccination status. Children in households with internet access had a full vaccination rate of 30.4%, versus 14.3% for those without access. However, this association was not statistically significant after adjustment (AOR: 1.31, 95% CI: 0.69–2.51). Children whose mothers who owned a mobile phone had a vaccination rate of 26.8%, compared to 16.0% among those who did not, though this was not significant after adjustment (AOR: 0.84, 95% CI: 0.52–1.35).

Children whose fathers had secondary education had a vaccination rate of 33.2%, compared to 16.3% among those whose fathers had no education. This was associated with significantly higher odds of being vaccinated (OR: 2.62, 95% CI: 2.19–3.13). Children living in households with only one child under five had a vaccination rate of 33.3%, while those in households with two or more children under five had a lower rate of 27.5%. Having two or more children under 5 significantly decreased the odds of being fully vaccinated (AOR: 0.20, 95% CI: 0.12–0.34). Children from rich households had a vaccination rate of 38.5%, compared to 27.5% among middle-income and 22.1% among poor households. Children from rich households had significantly higher odds of being fully vaccinated (AOR: 2.09, 95% CI: 1.14–3.83). Urban children were more likely to be fully vaccinated (40.9%) compared to rural children (24.7%). This association remained significant after adjustment (AOR: 0.70, 95% CI: 0.51–0.94).

## Discussion

This study aimed to identify the prevalence and associated factors of full vaccination among children aged 12–35 months in Yemen during internal conflict using data from MICS 2022-23. Overall vaccination coverage was low at 29%, with notable differences between urban (41%) and rural (25%) areas. While no MICS surveys were conducted in Yemen between 2006 and 2022–23, the most recent pre-conflict nationally representative survey, the 2013 Yemen National Health and Demographic Survey (YNHDS), reported 36% vaccination coverage among children aged 12–23 months (Ministry of Public Health and Population (MOPHP), 2015), while this figure in MICS 2022-23 was 30%, indicating a significant decline in post-conflict vaccination coverage. Notably, no pre-war benchmarks exist for children aged 24–35 months or the combined 12–35-month cohort analyzed here. Our study, focusing on children aged 12–35 months during a protracted conflict, found a lower coverage rate of 29%, with urban (41%) and rural (25%) disparities. While these findings provide a post-conflict benchmark, direct comparability to pre-war estimates is limited due to differences in survey timing, age-group definitions, and the severe degradation of Yemen’s health infrastructure since 2015. Despite these constraints, by applying multilevel logistic regression, we move beyond descriptive trends to quantify how maternal education, prenatal care utilization, household wealth, and media exposure individually and jointly influence full-vaccination uptake in Yemen’s war-affected setting. These insights can guide the design of targeted, evidence-based interventions to strengthen immunization delivery under conflict conditions.

While direct comparisons cannot be made, the low vaccination rates in Yemen likely lag behind many neighboring countries in the region. Many neighboring Gulf countries like Saudi Arabia, UAE, and Oman tend to have much higher vaccination rates above 90% for most vaccines (WHO, 2023). Before the war, Yemen had a vaccination rate reaching 70–80% of the target population. However, vaccination coverage dropped remarkably after the war began in 2015. The drop in coverage was more pronounced in governorates that witnessed armed confrontations like Taiz, Lahj, and Sa’dah (Torbosh et al., 2019). The decline in vaccination rates led to a surge in vaccine-preventable diseases (VPDs) like measles, cholera, and diphtheria after the start of the war (El Bcheraoui et al., 2018). The risk of polio importation also remains a major threat to Yemen and neighboring countries due to the low vaccination coverage amidst the conflict situation (Torbosh et al., 2019). The urban-rural divide underscores the substantial challenges in delivering immunization services equitably across regions with different levels of urbanicity and access to healthcare facilities (Belt et al., 2023). In many low-middle-income countries (LMICs) like Ethiopia, India, Myanmar, Pakistan, and Vietnam, vaccination coverage tends to be higher in urban areas compared to rural areas (Ali et al., 2022; Restrepo-Méndez et al., 2016). This is likely due to better access and proximity to healthcare facilities in urban centers (Restrepo-Méndez et al., 2016). However, in some countries like China, Gambia, Nigeria, and Zambia, the opposite trend is observed, with higher vaccination rates in rural areas. This could be due to targeted interventions or outreach programs reaching rural populations more effectively (Ali et al., 2022). Thus, efforts to improve vaccination coverage in Yemen’s rural areas should focus on enhancing healthcare access and delivery.

Higher maternal education levels were significantly associated with increased vaccination rates, not only in our conflict-era analysis but also in Yemen’s pre‐war surveys. The 2006 MICS report identified maternal secondary or higher education as a key correlate of BCG, DTP, and measles uptake (Ministry of Health and Population, 2008), and the 2013 DHS showed that children of mothers with secondary or higher education had approximately 1.5-times greater odds of full immunization compared to those whose mothers had no formal schooling (Ministry of Public Health and Population (MOPHP), 2015). In our study, children of mothers with secondary or higher education had higher odds of being fully vaccinated. This finding aligns with previous research suggesting that educated mothers are more likely to understand the importance of vaccinations and have better access to healthcare services (Forshaw et al., 2017; Hazan et al., 2019). Similarly, higher education levels of fathers were associated with increased vaccination rates, indicating that paternal education also plays a critical role in health decision-making within households (Rammohan et al., 2012). Notably, although the odds ratios for paternal and maternal education were comparable, the authority vested in male heads of household in Yemen likely amplifies the practical impact of paternal education on vaccine uptake (Bamatraf & Jawass, 2018). Only 55% of Yemeni women report participating in decisions related to their own health care (Ministry of Public Health and Population (MOPHP), 2015), with husbands or male relatives predominantly driving these choices. Thus, educated fathers may be better positioned to access information and authorize or prioritize vaccinations for their children (Bamatraf & Jawass, 2018), underscoring why paternal education emerges as a critical lever for improving immunization coverage. Therefore, while maternal education and involvement are crucial, this study underscores paternal education’s significant role in influencing household vaccination decisions. Consequently, efforts to improve health literacy and education, particularly among fathers, may help address disparities in childhood vaccination rates in Yemen.

Receiving prenatal care was strongly associated with higher vaccination rates in this study. Previous studies have shown that mothers who attended regular prenatal appointments were significantly more likely to ensure their children received recommended vaccinations on schedule (Triunfo et al., 2023). This underscores the value of leveraging the prenatal care setting as a platform to provide expecting mothers with accurate information about the importance, safety, and effectiveness of childhood vaccinations. Integrating childhood vaccination counseling and services as a routine component of prenatal care can help boost compliance and ensure more children receive recommended vaccinations on schedule to safeguard their health (Navar et al., 2007; Triunfo et al., 2023).

Exposure to media, particularly newspapers, was associated with higher vaccination rates in this study. This finding must be interpreted in the context of overall female literacy: only 50.8% of women age 15–49 years in Yemen were classified as literate (71.5% in urban vs. 41.0% in rural areas), which helps explain why just 6.9% of mothers reported reading newspapers at least weekly (Yemen, 2022−23). In Yemen, newspapers remain a trusted source among literate adults for health information, often carrying government and NGO vaccination campaigns; regular readers may therefore be more aware of immunization schedules and benefits (Atemthi Dau, 2023). While watching TV showed higher vaccination rates, the association was not significant after adjustment. It is important to note that only 51.8% of households owned a television set (77.8% urban vs. 39.8% rural), which constrains the reach of TV-based messages (Yemen, 2022−23). While the results do not directly compare newspapers vs. TV, one could infer that newspapers, being a more curated and edited medium, may provide more accurate and positive information about vaccines compared to TV, which can have more variable quality of health information (Recio-Román et al., 2023). However, the impact of different media types likely depends on factors like the specific content, framing, perceived credibility of the source, and accessibility/reach among different population groups (Haase et al., 2015; Melović et al., 2020). Nonetheless, it is difficult to definitively conclude that newspapers are more effective than TV in improving vaccination rates through health communication campaigns. More research directly comparing the influence of different media types and their specific content would be needed to support that claim.

Children from wealthier households were more likely to be fully vaccinated in conflict-era analysis, mirroring trends observed in Yemen’s non‐conflict periods. For example, the 2006 MICS report documented that children in the highest wealth quintile had nearly twice the full‐immunization coverage of those in the poorest quintile (Ministry of Health and Population, 2008), and the 2013 DHS similarly found that children from rich households had nearly 2‐fold greater odds of completing the vaccination schedule compared with those from poor households (Ministry of Public Health and Population (MOPHP), 2015). Economic stability likely provides better access to healthcare services and enables families to prioritize their children’s health needs (Hill, 2023; Quilici et al., 2015). A previous multi-country study indicated that higher GDP per capita and life expectancy at birth were associated with greater economic and social benefits from childhood vaccination programs across the BRICS countries (Brazil, Russia, India, China, and South Africa) (Mirelman, et al., 2014). Another important finding in this study was the lower likelihood of being fully vaccinated among children living in households with two or more children under five. This may be due to the logistical and financial barriers to getting all children fully vaccinated in households with multiple young children. Parents with several young children may face greater difficulties in scheduling and attending multiple vaccination appointments, especially if they have work or other commitments (Guerra, 2007). Additionally, while childhood vaccines are generally covered by insurance or government programs, there can still be associated costs like co-pays, transportation, and missed work hours. These costs may add up for households with multiple children needing vaccinations (Davis et al., 2002). Therefore, policies in Yemen should aim to alleviate these burdens and facilitate vaccination for larger families. This could be achieved by offering flexible vaccination appointment times and locations, providing incentives or support for parents (e.g., paid time off, childcare assistance), and implementing reminder/recall systems and follow-up mechanisms.

### Limitations

Although this study is the first to portray vaccination coverage among children aged 12–35 months in Yemen during the conflict, there are several limitations to consider when interpreting the results. The cross-sectional design limits causal inferences, as it provides only a snapshot of the situation. Self-reported data on immunization status can introduce recall and social desirability biases. Selection bias is possible as a result of inaccessibility to 41 enumeration areas during fieldwork due to conflict, which may affect the representativeness of the sample. Some confounding variables, such as cultural beliefs and community-level influences, were not analyzed due to a lack of data. Besides, predictor selection was guided by a backward stepwise logistic regression which can risk overfitting, biased estimates, and inflated Type I error (Henderson & Denison, 1989); however, to mitigate these, we also retained variables based on strong theoretical relevance—for example, internet access, mobile phone ownership, and TV viewing—based on prior evidence of their association with vaccination coverage (Geweniger & Abbas, 2020; Williams et al., 2024). Even though geographic disparities were highlighted, specific infrastructural challenges in rural areas were not detailed. The dynamic nature of the ongoing conflict in Yemen since 2015 is not fully captured by the study’s data. Additionally, the study does not assess the effectiveness of specific interventions aimed at improving immunization rates. Addressing these limitations in future research could provide a more comprehensive understanding of childhood immunization coverage in Yemen as a conflict-affected zone.

Despite its limitations, this study offers several notable strengths. It leverages recent, nationally representative data from the Yemen MICS 2022-23, a critical resource in conflict-affected settings where reliable health metrics are scarce. The use of multilevel logistic regression robustly accounts for clustering effects at the maternal level, enhancing the validity of associations identified. The analysis incorporates a comprehensive range of sociodemographic determinants, including maternal and paternal education, prenatal care utilization, media exposure, and household wealth, providing nuanced insights into immunization barriers. By focusing on children aged 12–35 months—a group at heightened risk of delayed or missed booster doses—the study addresses a critical gap in conflict-affected contexts.

## Conclusion

This study reveals significant gaps in immunization coverage among children aged 12–35 months in Yemen, particularly in rural areas. These gaps may be influenced by parental education, household wealth, healthcare access, and geographic location. To address these gaps, policymakers should implement educational programs for both mothers and fathers, enhance healthcare infrastructure, and improve health communication strategies. Tailored interventions such as mobile vaccination units and community health worker initiatives are recommended to reach marginalized populations. Future research should employ longitudinal designs to establish causal relationships, verify vaccination records for accuracy, explore infrastructural challenges in rural regions, and evaluate the effectiveness of specific interventions. Additionally, continuous monitoring of the dynamic conflict situation is essential to ensure resilient and equitable immunization coverage.

## Data Availability

The Yemen MICS 2022-23 dataset is publicly available on UNICEF’s official website through the following link: https://mics.unicef.org/surveys.
